# Supplementary material for: An open-source framework for large-scale, flexible evaluation of biomedical text mining systems
Source: J Biomed Discov Collab. 2008 Jan 29;3:1. doi: 10.1186/1747-5333-3-1 (PMC2276192; doi:10.1186/1747-5333-3-1)
Supplement: Additional file 1 — Evaluation platform source code. The additional file contains the software evaluation framework discussed in this paper. The most current release can be downloaded from SourceForge.net [1]. See the accompanying README file for instructions on installation and use. Also included in the distribution is a collection of UIMA wrappers for some commonly used BioNLP tools and annotated corpora. [file 1747-5333-3-1-S1.zip › bionlp_uima_repository-1.0.1/docs/api/edu/uchsc/ccp/uima/ae/nlp/sentenceDetector/package-frame.html]

edu.uchsc.ccp.uima.ae.nlp.sentenceDetector (Center for Computational Pharmacology Util/UIMA API)


edu.uchsc.ccp.uima.ae.nlp.sentenceDetector

Classes 
  
KeXSentenceDetector\_AE
  
LingPipeSentenceDetector\_AE
  
OpenNLPSentenceDetector\_AE
